# Syncope: epidemiology, etiology, and prognosis

**DOI:** 10.3389/fphys.2014.00471

**Published:** 2014-12-08

**Authors:** Rose M. F. L. da Silva

**Affiliations:** Department of Internal Medicine, Faculty of Medicine, Federal University of Minas GeraisBelo Horizonte, Brazil

**Keywords:** vasovagal syncope, cardiac arrhythmias, orthostatic intolerance, epidemiology, prognosis

## Abstract

Syncope is a common medical problem, with a frequency between 15% and 39%. In the general population, the annual number episodes are 18.1–39.7 per 1000 patients, with similar incidence between genders. The first report of the incidence of syncope is 6.2 per 1000 person-years. However, there is a significant increase in the incidence of syncope after 70 years of age with rate annual 19.5 per thousand individuals after 80 years. It presents a recurrence rate of 35% and 29% of physical injury. Among the causes of syncope, the mediated neural reflex, known as neurocardiogenic or vasovagal syncope, is the most frequent. The others are of cardiac origin, orthostatic hypotension, carotid sinus hypersensitivity, neurological and endocrinological causes and psychiatric disorders. The diagnosis of syncope can be made by clinical method associated with the electrocardiogram in up 50% of patients. Its prognosis is determined by the underlying etiology specifically the presence and severity of cardiac disease. The annual mortality can reach between 18 and 33% if cardiac cause, and between 0 and 12% if the non-cardiac cause. Thus, it is imperative to identify its cause and risk stratification for positive impact in reducing morbidity and mortality.

## Definition and epidemiology

Syncope is the sudden loss of consciousness, associated with inability to maintain postural tone, with immediate and spontaneous recovery without requiring electrical or chemical cardioversion. This framework is secondary to cerebral hypoperfusion, with short duration (average 12 seconds). It has a prevalence of 42%, considering a life time of 70 years and an annual incidence of 6% (Moya et al., 2009). Its frequency varies from 15% (below 18 years of age) (Lewis and Dhala, 1999) to 39% (among medical students) (Serletes et al., 2006), reaching 23% among the elderly (Lipsitz et al., 1985). In the general population, the annual number episodes are 18.1–39.7 per 1000 patients, with similar incidence between genders, and with high prevalence between 10 and 30 years of age, mainly of vasovagal syncope (Moya et al., 2009). The first report of the incidence of syncope is 6.2 per 1000 person-years. However, there is a significant increase in the incidence of syncope after 70 years of age, with 5.7 episodes/1000 individuals per year between 60 and 69 years old and with 11.1 episodes/1000 individuals per year between 70 and 79 years age. After 80 years, the annual incidence may reach 19.5 per 1000 individuals (Soteriades et al., 2002; Colman et al., 2004).

This framework is responsible for 3–5% of emergency department visits, with a hospitalization rate in about 40% of cases, with an average stay of 5.5 days. Beyond this morbidity, there is a recurrence rate of approximately 35% and 29% of physical injury, and major trauma in 4.7% of patients. In elderly patients with syncope due to carotid sinus hypersensitivity, the prevalence of trauma may reach 43% (Brignole et al., 2006; Moya et al., 2009). Besides the social impact with worsening quality of life, there is also the economic impact, with higher costs attributed to hospitalization with an estimated $2.4 billion annual cost (Sun, 2013).

## Etiology

Among the causes of syncope, the mediated neural reflex, known as neurocardiogenic or vasovagal syncope, is the most frequent, accounting for one third of the causes and reaching 66% of cases in emergency units (Alboni et al., 2001; Brignole et al., 2006). The diagnosis of syncope can be made by clinical method associated with the electrocardiogram in up 50% of patients (Moya et al., 2009). The clinical history should be performed with proficiency, and each episode of syncope should be well characterized about presence of prodrome, precipitating factors, conditions in which it occurred, position of the patient, and other associated signs such as nausea, pallor, diaphoresis, muscle twitching, confusion, physical injury, palpitations, dyspnea, chest pain, and cyanosis. The personal medical history and family history are also important to identify the cause of syncope. The Calgary Score is one of the diagnostic tools, including seven clinical issues and allow diagnosis vasovagal syncope with 89% sensitivity and 91% specificity (Sheldon et al., 2006). The physical examination should be comprehensive, including measurement of blood pressure in the supine position and within 3 min in the orthostatic position, the blood pressure measurements in both arms (to detect subclavian steal), and the exam of the cardiovascular system, which can identify signs of disease and/or arrhythmias, and other systems.

The main causes of syncope are summarized in Table 1 and will be discussed below.

**Table 1:** **Common causes of syncope**.

**Pathophysiological origin**	**Causes**
Reflex syncope	Vasovagal, situational syncope, orthostatic hypotension, carotid sinus hypersensitivity
Cardiac syncope	Structural heart disease, bradyarrhythmias, tachyarrhythmias
Neurological causes	Cerebrovascular disease, autonomic dysfunction, subclavian steal syndrome
Others causes	Endocrinological causes, psychiatric disorders

### Vasovagal syncope

This term was first used by William Gowers in 1907 and described the mechanism of vasovagal syncope by Thomas Lewis in 1932 (Lewis, 1932). It shows bimodal distribution, but is most common in young people. Precipitating factors are prolonged sitting position or standing position, emotional stress, pain, heat, venous puncture, alcohol use, dehydration, use of diuretics and vasodilators. Prodromes of nausea, vomiting, abdominal pain, diaphoresis, pallor, palpitations, and dizziness may occur and are more common in young people. After loss of consciousness, tonic-clonic contractions, if they occur, are of short duration (<15 s). There are no aura, headache, sleep, sphincter release, mental confusion, and duration of loss of consciousness greater than 5 min, such as occurs in epilepsy (Kapoor, 2002; Moya et al., 2009). The neuromediated syncope is called situational syncope when it occurs in conditions that trigger the Valsalva maneuver, such as urination, defecation, coughing, visceral pain, carry weight.

The mechanism of vasovagal syncope is explained by the Bezold–Jarisch reflex, which is triggered due to decreased venous return resulting in inadequate ventricular filling and vigorous cardiac contraction. It occurs by the action of mechanoreceptors (C fibers) preferentially located in the inferolateral wall of the left ventricle, but also in the atria and in the pulmonary artery, and manifests with hypotension and paradoxical bradycardia due to increased activity of inhibitory receptors and consequent parasympathetic hyperactivity (Medow et al., 2008; Moya et al., 2009).

Initial therapy of vasovagal syncope is non-pharmacological. The recommendations are to identify and avoid the precipitating factors, assuming the supine position during the period of prodromes, and nutritional guidelines. These include hydration with fluid intake 2–2.5 liters per day and sodium supplementation. Aerobic and isometric exercises of the upper and lower limbs reduce the recurrence of vasovagal syncope. Tilt training compared with conventional treatment shows limited efficacy in controlled studies. The use of compression stockings in the lower limbs may be recommended, however it has limited efficacy and poor adherence. If failure of initial recommendations, the next step is pharmacological treatment with the use of fludrocortisone if vasodepressor response. Beta blockers are contraindicated. Implantation of a pacemaker is class IIa indication for patients over 40 years of age and cardioinhibitory response with asystole (Moya et al., 2009; Raj and Coffin, 2013).

### Cardiac causes

The causes are structural heart diseases or conditions that result in decreased cardiac output such as aortic stenosis, hypertrophic cardiomyopathy, ischemic heart disease, heart failure, aortic dissection, cardiac tamponade, prosthetic valve thrombosis, cardiac tumors, pulmonary hypertension, pulmonary embolism etc. Both bradyarrhythmias and tachyarrhythmias are frequent causes of cardiac syncope due to impaired cardiac output secondary to the frequency of the arrhythmia, its origin, to systolic ventricular dysfunction. A family history of sudden cardiac death is an important data for the hypothesis channelopathy (Moya et al., 2009; Rosanio et al., 2013). Treatment depends on the cause cardiac syncope, with treatment directed to the structural heart disease, pacemaker implantation, if bradyarrhythmias, or use of antiarrhythmic, catheter ablation or implantable cardioverter-defibrillator, if tachyarrhythmias (Moya et al., 2009).

### Orthostatic hypotension

Orthostatic or postural hypotension (OH) presents as falls, dizziness, or syncope, resulting in functional impairment, with head injury, bone fractures and hospitalization. It is more common in the elderly, with frequency rates of up to 55% in those that residing in institutions, and with prevalence of 12%. Its classic definition is a drop of at least 20 mmHg in systolic and/or 10 mmHg in diastolic blood pressure within 3 min to take an orthostatic position. There are other presentations such as initial OH, when there is a greater than 40 mmHg drop in blood pressure, with less than 30 s in duration, and progressive OH, when the fall of blood pressure levels are gradual, between 3 and 30 min after taking standing position, without bradycardia (Goldstein and Sharabi, 2009; Moya et al., 2009).

Beyond the control of the precipitating factor, hydration and salt intake, treatment of syncope postural hypotension can be done with fludrocortisone, midodrine. Other measures are abdominal compression and elevation of the head of the bed (Moya et al., 2009; Raj and Coffin, 2013).

Postprandial hypotension is a common cause of syncope in the elderly, with a prevalence that can reach 67%, especially in the elderly who live in institutions. It is defined as the drop of at least 20 mmHg in systolic blood pressure or absolute value of the systolic pressure lower than 90 mmHg (those with systolic blood pressure of at least 100 mmHg), within 2 h after meals. The pathophysiology is due to sympathetic dysfunction, with inadequate peripheral vasoconstriction and insufficient heart rate increase. Precipitating factors are vasodilators, high temperature of food or of environment and diets high in carbohydrates (Luciano et al., 2010).

### Carotid sinus hypersensitivity

It is an extrinsic sinus node disease that is characterized by pre-syncope or syncope exacerbated by the carotid sinus reflex response. Its incidence is 35–40 patients/year/million individuals, with a predominance in males (male:female ratio of 4:1) and more frequent in the elderly, especially diabetics with coronary or carotid atherosclerosis. Precipitating factors are sudden movements of the head and neck, cervical compressions and use of tight neck tie (Healey et al., 2004). The approach of the carotid sinus syndrome includes the implantation of a pacemaker if cardioinhibitory response, or use of volumetric expansion, if vasodepressor response.

### Neurological causes

Neurological causes are cerebrovascular disease, autonomic dysfunction and subclavian steal syndrome. Focal neurological deficits in stroke, vertebrobasilar transient ischemic stroke, migraine (for vasospasm or vasovagal reflex) may be presented as syncope.

The primary autonomic dysfunction occurs in pure primary dysfunction syndrome (Bradbury–Eglleston), in central nervous system diseases (Parkinson's disease, multiple system atrophy or Shy–Drager syndrome, Huntington's disease and Guillain–Barré syndrome). Secondary dysfunctions occur by changes of aging, due to the involvement of the peripheral nervous system in diabetes mellitus, renal failure, alcoholism, amyloidosis; infections of the nervous system by Chagas disease, human immunodeficiency virus; metabolic diseases such as vitamin B12 deficiency, porphyria; autoimmune diseases such as rheumatoid arthritis and others. And as antihypertensive drugs (diuretics, vasodilators), anti-depressants may also cause autonomic dysfunction (Azhar and Lipsitz, 1998).

The first report of subclavian steal syndrome was described in 1960 by Contorni. It shows a prevalence of up to 6.4%. There is malformation or obstruction by atherosclerosis of the proximal subclavian artery to the origin of vertebral artery, resulting in retrograde flow in this artery. It occurs mainly in the left subclavian artery. Neurological symptoms, such as dizziness, paresthesia and syncope, occur during exercise performed by the arm, but patients may present framework of transient ischemic attacks (Osiro et al., 2012; Potter and Pinto, 2014).

### Others causes

There are endocrinological causes that can be presented as orthostatic hypotension to cause autonomic dysfunction or hypovolemia. As examples, chronic adrenal insufficiency and hypopituitarism can be cited. Diabetes insipidus and salt-losing nephropathies since they result in volume depletion, and pheochromocytoma and carcinoid syndrome, due to vasoactive substances, can also be causes of syncope. Hyperventilation and psychiatric disorders, due to cerebral hypoperfusion, are other causes of syncope (Kapoor, 2002; Moya et al., 2009).

## Prognosis

The pathophysiology, approach, prognosis and treatment depend on the cause of syncope, and mandatory their identification, since their annual mortality can reach between 18 and 33% if cardiac cause, and between 0 and 12% if the non-cardiac cause (Kapoor, 2002).

There are studies that compared mortality among patients with syncope of cardiac origin and non-cardiac origin. Among participants in the Framingham heart Study, 7814 patients were included from 1971 to 1998 and 822 presented syncope of which 9.5% were cardiac. Adjusted multivariate analysis showed that the risk of death increased by 31% among all patients with syncope and was doubled among those with syncope of cardiac origin, compared with those without syncope. Neurological cause of syncope was also associated with a threefold risk of stroke. Moreover, there was no association with death or major adverse events among those with vasovagal syncope (Soteriades et al., 2002).

In the study by Ungar et al. with 380 patients included, death from any cause occurred in 35 (9.2%) patients during the mean follow-up of 614 days. Death was considered cardiovascular in 9 patients (26%). And among the patients who died, 82% were older and had cardiac risk factors such as abnormal ECG and/or heart disease. In contrast, among those with no abnormal ECG and/or heart disease, only six (3%) deaths occurred, resulting in a negative predictive value of 97% (Ungar et al., 2010).

Another study of 200 patients with syncope, cardiac origin was associated with adverse events defined as death, recurrence of syncope, cardiovascular events, and major procedures during the following short-term (1 month) and long (1 year) (Numerosos et al., 2013).

A study of 37,017 patients with syncope in the period 2001–2009, without previous hospitalization for comorbidities, and with a control group of 185,085 individuals, demonstrated increased risk of all-cause mortality, stroke, cardiovascular hospitalization, device implantation, and recurrent syncope in healthy individuals after first admission for syncope (Ruwald et al., 2013). Another study more recent from the same team, with 70,819 patients hospitalized between 2001 and 2009, aged between 19 and 90 years, with a mean follow up of 3.9 years, showed that recurrence of syncope, which occurred in 16.4%, was associated to 3.2 times the risk of death within a year (Ruwald et al., 2014).

Regarding the recurrence of syncope, risk stratification cannot predict it. The incidence of recurrence of syncope was similar regardless of cause of syncope. Its rate was 0.3% in the first month, of 0.8% per month during the first year and 0.5% per month during the second year. On univariate analysis, predictors of recurrence were male sex, presence of prodrome and absence of palpitations (Ungar et al., 2010). On the other hand, the number of vasovagal syncope events in the preceding year may be a predictor of recurrence. Those patients with less than two previous episodes, the recurrence was 22% vs. 69% those with more than 6 episodes, with a probability of 46% (Sumner et al., 2010).

Thus, prognosis is determined by the underlying etiology specifically the presence and severity of cardiac disease. And it is imperative to identify its cause and risk stratification for positive impact in reducing morbidity and mortality.

### Conflict of interest statement

The author declares that the research was conducted in the absence of any commercial or financial relationships that could be construed as a potential conflict of interest.
